# New myrmecomorphous longhorned beetles from Haiti and the Dominican Republic with a key to Anaglyptini and Tillomorphini of Hispaniola (Coleoptera, Cerambycidae, Cerambycinae)

**DOI:** 10.3897/zookeys.106.1470

**Published:** 2011-06-15

**Authors:** Steven W. Lingafelter

**Affiliations:** Systematic Entomology Laboratory, Plant Sciences Institute, Agriculture Research Service, U.S. Department of Agriculture, National Museum of Natural History, Washington, D.C. 20013-7012, U.S.A.

**Keywords:** Island, endemic, mimicry, myrmecophily, West Indies, taxonomy

## Abstract

First records of the tribes Anaglyptini and Tillomorphini (Coleoptera: Cerambycidae: Cerambycinae) are documented for Hispaniola. A new genus of a highly myrmecomorphic longhorned beetle (*Licracantha* **gen. n.**) is described and illustrated based on one species (*Licracantha formicaria* **sp. n.**) and provisionally assigned to Tillomorphini. Three other new species of ant mimic longhorned beetles are described and illustrated: *Calliclytus macoris* **sp. n.** (Tillomorphini), *Tilloclytus baoruco* **sp. n.**, and *Tilloclytus neiba* **sp. n.** (Anaglyptini). An identification key and distribution map to all known Hispaniolan species of these two tribes are presented.

## Introduction

Hispaniola is among the most critical biodiversity regions in the world and is part of the Caribbean Islands Hotspot (Conservation International and McGinley 2008). The entomological riches there were first discovered and described by Palisot de Beauvois (1805–1821). Despite periods of intense beetle collecting in the late 19th, early 20th, and early 21st centuries, many species of Hispaniolan Cerambycidae still remain undescribed. In the last 5 years, 20 new species representing about 12% of the known fauna have been described, demonstrating how poor our prior knowledge was (Lingafelter 2008, 2010; Lingafelter and Micheli 2009; Lingafelter and Nearns 2007; Lingafelter and Woodley 2007).

Batesian mimicry has been well documented in longhorned beetles and has been summarized in Shelford (1902) and Linsley (1959). The genera in the tribe Tillormorphini Pascoe [and some in Anaglyptini Lacordaire] are considered as group mimics of ants with generalized structural modifications (Linsley 1959). Morphological adaptations that suggest an antlike facies include a constriction of the elytra around the middle, accentuated with a light colored fascia or ivory colored callus (representing a petiole) and an inflated pronotum (representing a large ant head when combined with the smaller, actual head) (McIver and Stonedahl 1993).

Studies such as Fisher (1932), Micheli (2003), Chalumeau and Touroult (2005), and Micheli (2010) have documented the species diversity in the West Indies for Anaglyptini and Tillomorphini, nearly all of which display varying degrees of myrmecomorphy. Several species have been documented as collected with ants in Puerto Rico and Hispaniola, suggesting that their mimicry of, and association with ants may provide a selective protection (Micheli 2003; Micheli 2010; and herein).

Prior to this work, the tribes Anaglyptini and Tillomorphini were unknown for Hispaniola (Perez-Gelabert 2008; Monné and Bezark 2010) [note that the genus *Hormathus* Gahan was previously placed in Tillomorphini but transferred to Ibidionini by Lingafelter and Nearns 2007]. The authorship of Tillomorphini and Anaglyptini was corrected in Bousquet et al. (2009): Tillomorphini was proposed by both Pascoe and Lacordaire in 1869, but Pascoe takes precedence since he published first; Anaglyptini was proposed by Lacordaire and the publication date was determined to be 1868 despite the 1869 date imprint on the title page.

In addition to the nomenclatural confusion, these tribes are very difficult to differentiate from each other (and Clytini) because they were never clearly defined and the currently recognized diversity in each tribe has escalated beyond their original, meager definitions. For example, the type genus of Anaglyptini, *Anaglyptus* Mulsant, has subsequently been considered a clytine (Gressitt 1951; Löbl and Smetana 2010), while some authors retained it as a separate tribe based on the presence of a mesal spine on antennomere 3 and lack of carinae or grooves on the frons (Bíly and Mehl, 1989). Adding to the confusion, the type species of Tillomorphini, *Tillomorpha lineoligera* Blanchard, has very coarsely faceted eyes and lacks a transverse ivory-like ridge or fascia on the elytra, unlike most of the species currently placed in that tribe (Lingafelter and Nearns 2007). Despite this, Pascoe (1869) used finely faceted eyes as the main feature to place Tillomorphini in the “second section” of Cerambycidae. Aurivillius (1912) listed *Tilloclytus* Bates and *Tillomorpha* Blanchard next to each other in the tribe Tillomorphini. However, Linsley (1964) placed *Tilloclytus* in the tribe Anaglyptini based on some characters he ascribed to Anaglyptini such as: head large; pronotum constricted at base; prosternum with intercoxal process narrow; and elytra gibbose at base and lacking transverse, ivory-like ridges. Zayas (1975) returned the six Cuban species of *Tilloclytus* to Tillomorphini (along with *Calliclytus* Fisher and *Pentanodes* Shaeffer), perhaps correctly so, but without explanation. The most current catalog of the Cerambycidae of the Neotropical Region (Monné 2005) followed Linsley (1964) and returned *Tilloclytus* to Anaglyptini, leaving all other West Indian tillomorphine genera mentioned by Chalumeau and Touroult (2005), Micheli (2010), and Zayas (1975) in the Tillomorphini. Thus, currently in the West Indies there is only one genus (*Tilloclytus*) in Anaglyptini and six genera (*Arawakia* Villiers, *Bonfilsia* Villiers, *Calliclytus* Fisher, *Gourbeyrella* Lane, *Lamproclytus* Fisher, and *Pentanodes* Schaeffer) in Tillomorphini (Monné and Bezark, 2010).

A spectacularly myrmecomorphic, monotypic new genus and new species (*Licracantha formicaria*) is described and provisionally placed in the tribe Tillomorphini. A new species of *Calliclytus* (*Calliclytus macoris*, sp. n.) is also described in this tribe. Two other species of Hispaniolan ant mimic Cerambycidae (*Tilloclytus baoruco*, sp. n. and *Tilloclytus neiba*, sp. n.) are described in the tribe Anaglyptini. Species in *Tilloclytus* and *Lamproclytus* are known to have significant intraspecific variation in color, and Micheli and Hovore (2003) observed this when recognizing several synonymies of Puerto Rican species described by (Fisher (1932, 1935). While most of the new species described herein are based on one or a few specimens each, they possess structural features (in addition to color patterns) that are unique to each, further demonstrating their taxonomic status.

## Methods

The material examined in this study was collected by Michael Ivie, Edmund Giesbert, Eugenio Nearns, Derek Sikes, Michael Thomas, Barry Valentine, and Norman Woodley. Holotypes are deposited in the Smithsonian Institution (USNM) and the Museum of Entomology at the Florida State Collection of Arthropods (FSCA). Holotype images in the USNM are available online in the Smithsonian Primary Type database (Lingafelter et al. 2011).

The species in this study are represented by one or a few specimens each. Many specimens are in imperfect condition; therefore, careful digital paintings were deemed preferable to show the beetles in natural, lifelike postures and to display the diagnostic characters. These paintings were produced by Taina Litwak (Systematic Entomology Laboratory, USDA [hereafter SEL]) using Photoshop in Adobe Creative Suite 4 on a G5 Macintosh with OS X.5.8.

Species determinations were aided by examination of material from many collections and type image websites. Those websites that were particularly useful, holding holotypes of related species, included: AMNH (2011) (which has the holotype of *Tilloclytus minutus* Fisher); MCZC (2011) (which has the holotype of *Tilloclytus rufipes* Fisher); and Lingafelter et al. (2011) (which has the holotypes of *Tilloclytus bruneri* Fisher, *Tilloclytus cubae* Fisher, *Tilloclytus puertoricensis* Fisher, *Lamproclytus elegans* Fisher, *Lamproclytus oakleyi* Fisher, and *Calliclytus schwarzi* Fisher). The paper on the Cuban Cerambycidae of the Zayas collection by Nearns et al. (2006) was very useful since it provided photographs of the holotypes of *Tilloclytus elongatus* Zayas and *Tilloclytus pilosus* Zayas, leading to the discovery of a synonomy (Lingafelter and Nearns, in press).

Morphological terminology follows Lingafelter (1998). Measurements were made using Axiovision software on images taken with a Zeiss AxioCam HRc camera attached to a Zeiss Discovery V20 stereomicroscope with Sycop motorized zoom and focus control and a PlanApo S 1.0X objective.

### Collection acronyms used in this study include:

ACMTAmerican Coleoptera Museum, San Antonio, Texas, U.S.A. (J. Wappes)

EFGCEdmund F. Giesbert Collection (at FSCA), Gainesville, Florida, U.S.A. (M. Thomas, P. Skelley)

FSCAFlorida State Collection of Arthropods, Gainesville, Florida, U.S.A. (M. Thomas, P. Skelley)

USNMNational Museum of Natural History, Smithsonian Institution, Washington, DC, U.S.A. (S. Lingafelter)

WIBFWest Indian Beetle Fauna Project, Bozeman, Montana, U.S.A. (M. Ivie)

## Results and discussion

### Licracantha

Lingafelter gen. n.

urn:lsid:zoobank.org:act:923ACE0A-618A-47F6-A830-93E5F8F9C75B

http://species-id.net/wiki/Licracantha

Figs 1 2

#### Diagnosis.

No other genus of Tillomorphini or Anaglyptini has the type of modified antenna, pronotum, and elytron as is present in *Licracantha*. The combination of the following character states is unique to *Licracantha*: antenna myrmecomorphic, 11-segmented, with elongate scape, antennomeres 3–5 with pronounced mesal spines, antennomeres 6–11 abruptly shortened, antennomeres 3–11 articulated in a potentially opposing direction from scape; pronotum highly and abruptly elevated at anterior four-fifths, with acute, suprascutellar projection posteriorly; eye finely faceted and as single lobe anteroventral to antennal insertion, elytra gibbose basally and apically with depression at oblique, unraised, ivory fascia; tibiae each with single, curved spine (most pronounced on meso- and metatibia).

*Gourbeyrella*, *Tillomorpha*, *Bonfilsia*, *Arawakia*, *Pentanodes*, and *Tilloclytus* each lack antennal spines. Further, *Bonfilsia*, *Arawakia*, *Lamproclytus*, and presumably, *Calliclytus* each have 10-segmented antennae. *Calliclytus* and *Lamproclytus* are further differentiated since each have upper eye lobes (along with the larger, lower lobe), a very short scape, and a pronotum that is not elevated anteriorly. A few species in the relatively large, heterogeneous genus *Tilloclytus* are most similar to *Licracantha* in having a moderately, anteriorly elevated pronotum, a single finely faceted eye lobe, and an elytron moderately gibbous at base and apex and with some type of pale transverse or oblique fascia near mid length. *Tilloclytus minutus* Fisher has the most similar antenna possessing an elongate scape and very short antennomeres 6–11; however, the myrmecomorphic modifications are not as extreme: the scape is shorter, extending only to the anterior third of the pronotum, the abrupt articulation allowing an opposing orientation of remaining antennomeres from 3–11 is not present, and antennomeres 3–5 are, at most, dentiform mesally. *Tilloclytus minutus* is further differentiated by having the elytron and pronotum uniformly, confluently alveolate-punctate, lacking a narrow, well defined pale elytral fascia, having the pronotum not abruptly elevated anteriorly, and in lacking a well developed gibbosity on the elytral base and apex. *Tilloclytus bruneri* Fisher is similar in having a posterior suprascutellar pronotal projection, but it is not as developed or acute as in *Licracantha*. *Tilloclytus bruneri* is further differentiated by having a longer, unmodified, unspined antenna and a pronotal elevation and elytral gibbosity that are less developed. It also has a glossy integument lacking micropunctation.

#### Type species.

This genus is known only from *Licracantha formicaria* Lingafelter, described below.

#### Etymology.

A latinized composite noun, female gender, derived from the Greek “Likros” meaning horn and the Greek “Akantha” meaning thorn. *Licracantha* refers to the pronounced spines on the antennae.

#### Remarks.

This new genus is provisionally assigned to the tribe Tillomorphini. The definitions and boundaries of Anaglyptini and Tillomorphini are vague and troublesome, as discussed above, and each may contain a polyphyletic assemblage of taxa. A phylogenetic analysis of all the genera in these tribes is needed to develop a meaningful classification. Once those studies are completed, they may show that the genus *Tilloclytus*, to which *Licracantha* shows some similarities, should be returned to Tillomorphini from Anaglyptini, in which case Anaglyptini would not be present in the Caribbean Region.

### Licracantha formicaria

Lingafelter sp. n.

urn:lsid:zoobank.org:act:2C031E71-45D5-4FD7-9514-A28A8803D8DC

http://species-id.net/wiki/Licracantha_formicaria

Figs 1 2 Map 1

#### Diagnosis.

The single known species is recognized by the modified myrmecomorphic antennae with strong mesal spines on antennomeres 3–5, the suprascutellar projection of the pronotum, the matte integument with micropunctation throughout the dorsal surface, the dense, white pubescence on the sides of the meso- and metasternum, the purple hue on the apical two-thirds of the elytron, and the glossy integument ventrally and on the elytral epipleuron.

#### Description.

Male. 4.86 mm long; 1.37 mm wide at humeri. **Color:** Integument of head, pronotum, elytron, and legs mostly dark reddish brown with purple hue on apical two-thirds of elytron and legs; antenna dark reddish brown except for base of scape and antennomeres 3–5 which are lighter orange. **Head:** Matte, with micropunctation throughout and indistinct larger depressions; setae sparse, short, golden; gena short, produced anteriorly into acute tooth near base of mandible; frons short, broad, without evident frontal-genal ridge, anteclypeal sulcus, or interantennal groove or depression; eye with single small lobe present anteroventral to antennal tubercle; laterally as protuberant as pronotum; finely faceted; antennal tubercle weakly, gradually elevated; antenna 11-segmented, short, extending to middle of elytron; highly myrmecomorphic with elongate scape extending to nearly middle of pronotum; antennomere 2 short, angled, causing remaining antennomeres to be potentially articulated in opposing direction from scape; antennomeres 3–5 spinose apicomesally, lighter than remainder; antennomeres 6–11 abruptly shortened, shorter than scape; antennomeres 1–5 with sparse, elongate, golden setae; 6–11 with denser, appressed golden setae. Mandible yellow with piceous apex; terminal palpomeres broadly dilated. **Pronotum:** Matte, with uniform micropunctation throughout and interspersed, separate, shallow punctures; without calli or tubercles; distinctly longer than broad, 1.61 mm long, 1.04 mm wide (length/width = 1.55); anterior four-fifths abruptly elevated above constricted posterior fifth; base distinctly narrower than elytron; posteriorly produced into suprascutellar process at middle; sparsely pubescent with scattered, erect and appressed, short, golden setae, more dense at posterior margin of elevation. **Prosternum:** Densely, uniformly micropunctate, glabrous; prosternal process very narrow between procoxae; broadly expanded behind, closing procoxal cavities posteriorly; highly impressed anterior to procoxae; strongly downwardly curved anteriorly. **Elytron:** Matte, except for glossy, anterior epipleural region; uniformly micropunctate throughout; scattered, sparse, appressed, golden setae, mostly concentrated on basal third; dark reddish brown to piceous at basal third, separated from lighter purplish brown apical two thirds with oblique pale fascia that does not extend to suture; constricted and depressed near white fascia, gibbous at base and apex; elytral apex rounded to suture; 2.79 mm long, 0.67 mm wide (length/width = 4.16). **Scutellum:** Small, rounded posteriorly; short, golden setae present on middle. **Legs:** Femora short, stout, with strongly clavate apices on abruptly narrowed peduncles; metafemur not attaining elytral apex; tibiae straight, apically expanded, each with single, strong, curved tibial spine; sparsely pubescent with scattered golden setae. **Venter:** Mostly glossy, sparsely pubescent except on sides of posterior margin of metasternum and mesosternum which have dense, white pubescence, the former coinciding with white fascia of elytron; mesosternal intercoxal process about 3 times as broad as prosternal process, with small lateral projection into mesocoxa and middle notch receiving anterior projection of metasternum. Ventrite 1 most elongate; remaining ventrites successively shorter and elevated towards elytral apex; apex of fifth ventrite broadly rounded, without notch, sulcus, or other modification.

**Map 1.**
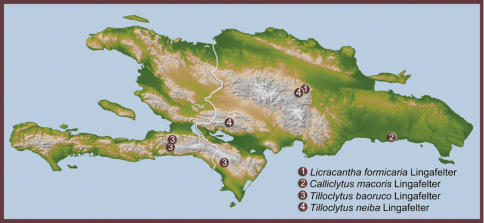
Distribution of ant-mimic longhorned beetles of tribes Tillomorphini and Anaglyptini in Hispaniola.

**Figure 1.**
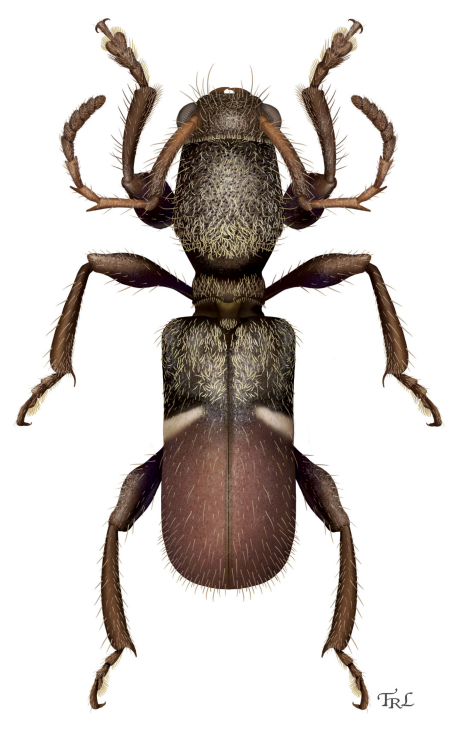
*Licracantha formicaria* sp. n., dorsal habitus. Digital painting by Taina Litwak.

**Figure 2.**
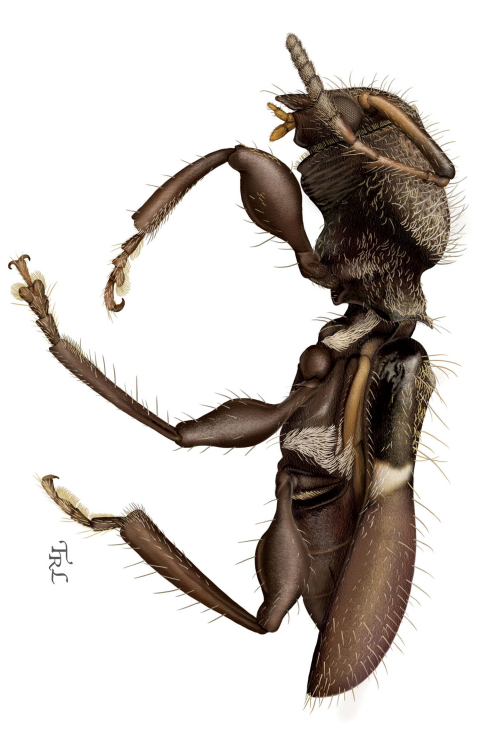
*Licracantha formicaria* sp. n., lateral habitus. Digital painting by Taina Litwak.

#### Etymology.

The specific epithet, *formicaria*, is a Latin adjective referring to the very antlike facies.

#### Type material.

Holotype, male: Dominican Republic, La Vega Prov., 4.7 km SE Jarabacoa, July 16, 1996, M. C. Thomas, collector (FSCA).

#### Remarks.

Only a single specimen is known of this monotypic genus. It is similar to arboreal ants of the genus *Cephalotes* Latreille, and they may be the model for this myrmecomorphic longhorn. These distinctive ants with large heads and spines on the thorax that resemble the suprascutellar process of the beetle, are slow moving and frequently beaten from vegetation in the Dominican Republic where this specimen was collected (Lingafelter pers. obs). There are six species of these ants known from Hispaniola (Perez 2008).

### Calliclytus macoris

Lingafelter sp. n.

urn:lsid:zoobank.org:act:CD9CE24D-72FB-4858-A16F-5D6DBDC55ACA

http://species-id.net/wiki/Calliclytus_macoris

Fig. 3 Map 1

#### Diagnosis.

This species is very similar to the Cuban *Calliclytus schwarzi* Fisher with regard to proportions, shapes of anatomical structures, position of the antemedial, raised, ivory callus of the elytron, and hypothesized presence of only 10 antennomeres (the holotype of *Calliclytus schwarzi* is missing the terminal segment of both antennae; however, since the antennal proportions are similar to those of *Calliclytus macoris*, it presumably has only 10 antennomeres). *Calliclytus macoris* differs from *Calliclytus schwarzi* in having an alveolate-punctate pronotum (rugose in *Calliclytus schwarzi*), a diamond shaped, pale macula at suture near elytral apex (elytral apex all black in *Calliclytus schwarzi*), and a densely pubescent scutellum (glabrous in *Calliclytus schwarzi*).

*Calliclytus macoris* is also similar to the Puerto Rican *Lamproclytus elegans* Fisher with regard to proportions and shapes of the major anatomical structures but differs in having the raised eburneous ridge of the elytron antemedially located (postmedially positioned in *Licracantha elegans*), the diamond shaped, pale macula at the elytral apex (uniformly dark in *Licracantha elegans*), and uniformly dark legs (femora pale at the base and dark at the apex in *Licracantha elegans*).

**Figure 3.**
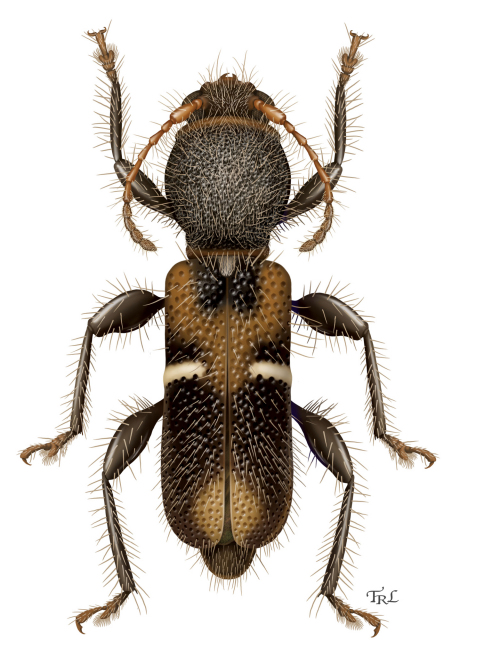
*Calliclytus macoris* sp. n., dorsal habitus. Digital painting by Taina Litwak.

#### Description.

Female. 4.85 mm long; 1.26 mm wide at humeri. **Color:** Dorsal integument of head, pronotum, and legs dark reddish brown to black; elytral color dark reddish brown to black on periscutellar region and most of the apical two-thirds with the exception of orange, diamond shaped macula at apex and raised, transverse ivory callus antemedially; ventral color mostly dark reddish brown to black except for orange head. **Head:** Shiny, rugose-punctate throughout; moderately dense, long and short erect and suberect, yellow-translucent setae; frons and gena short, broad, without acute projection near base of mandible; with poorly defined frontal-genal ridge; without anteclypeal sulcus; without interantennal groove or depression; eye divided into two lobes connected by row of 5 facets, with large lobe anteroventral positioned to antennal tubercle and small lobe present posterodorsal to antennal tubercle; laterally not as protuberant as pronotum; finely faceted; antennal tubercle weakly elevated; antenna 10-segmented, without spines, short, extending to just beyond base of elytron; scape short, thickened at middle, extending just past anterior margin of pronotum; antennomere 2 short, but over half length of antennomere 3; antennomeres 2–5 successively increasing in length, 6–10 successively shorter, produced apicolaterally; scape through antennomere 5 pale orange; 6–10 piceous to black; antennomeres 1–5 with sparse, elongate, golden setae; 6–10 with denser, appressed golden setae along with sparse, erect setae. Mandible short, retracted, yellow with piceous apex; terminal palpomeres not broadly dilated. **Pronotum:** Semiglossy, with uniform alveolate punctation dorsally, becoming punctate at sides; without calli or tubercles; slightly longer than broad, 1.47 mm long, 1.27 mm wide (length/width = 1.16); evenly widened at middle; gradually rounded laterally and dorsally; base with constriction; distinctly narrower than elytra; sparsely but conspicuously pubescent with scattered, long, erect yellowish setae combined with shorter, appressed yellow setae. **Prosternum:** Glossy, with dense microsculpture and short, white, setae in front of procoxae; prosternal process narrow between procoxae; apex broadly expanded behind, closing procoxal cavities posteriorly. **Elytron:** Glossy; deeply, separately punctate throughout, becoming slightly more dense posteriorly; sparse but conspicous, erect, yellow setae throughout; dark reddish brown to black on periscutellar region and most of the apical two-thirds with exception of orange, diamond shaped macula at apex; transverse, raised, eburneous callus present, not extending to suture; weakly gibbous at periscutellar region only; elytral apex rounded to suture; 2.96 mm long, 0.62 mm wide (length/width = 4.77). **Scutellum:** Elongate, subtruncate at posterior apex; densely coated with appressed, short, yellowish setae. **Legs:** Femora short, stout, with strongly clavate apices on abruptly narrowed peduncles; metafemur not attaining elytral apex; tibiae straight, not expanded apically, each with two straight tibial spines; tibiae and femora sparsely but conspicuously pubescent with long, erect, white setae. **Venter**: Glossy; sparsely pubescent throughout with erect, long, white setae and dense, short, white setae on metasternum posterior and lateral margin, mesosternum, and prosternum; integument darker than most of dorsum; mesosternal intercoxal process about 2.2 times as broad as prosternal process, without lateral projection into mesocoxa. Ventrite 1 most elongate; remaining ventrites much shorter and subequal in length; apex of fifth ventrite broadly rounded, without notch, sulcus, or other modification.

#### Etymology.

The specific epithet is based on the nearby Macorís River where this species was discovered by Edmund Giesbert.

#### Type material.

Holotype, female: Dominican Republic, San Pedro de Macorís Prov., 12 km W San Pedro de Macorís, May 5–19, 1985, E. Giesbert, collector (EFGC in FSCA).

#### Remarks.

The genera *Lamproclytus* and *Calliclytus* were not specifically compared to each other in Fisher’s (1932) descriptions, despite their obvious similarities in nearly every feature. Careful phylogenetic work in Tillomorphini may suggest that synonymy of these genera is necessary, but that is beyond the scope of this work. Given that the position of the eburneous elytral ridge of *Calliclytus macoris* is closest to that of *Calliclytus schwarzi*, it is placed in that genus as opposed to *Lamproclytus*. Note that Monné (2005) and Monné and Bezark (2010) erroneously listed *Lamproclytus elegans* Fisher from the Dominican Republic, but that species does not occur in Hispaniola.

This new species is superficially similar to ants of the genus *Leptothorax* Mayr, which may serve as the mimicry model. In Puerto Rico, the similarly colored cerambycid, *Boricyrtinus nilseni* Micheli, was collected with *Leptothorax isabellae* (Wheeler) (Micheli 2003). There are seven species of these ants known from Hispaniola (Perez 2008).

### Tilloclytus baoruco

Lingafelter sp. n.

urn:lsid:zoobank.org:act:8B4B5147-A091-4137-88FE-1D0EBC4CAC1E

http://species-id.net/wiki/Tilloclytus_baoruco

Fig. 4 Map 1

#### Diagnosis.

This species is unique among West Indian *Tilloclytus* in having only 10 antennomeres. It is otherwise most similar to *Tilloclytus bruneri* Fisher from Cuba in that the antemedial pale band of short, appressed pubescence is incomplete, not reaching the suture, but they are easily differentiated by color: *Tilloclytus baoruco* is mostly light brown to orange, while *Tilloclytus bruneri* is darker bluish black.

#### Description.

3.34–4.67 mm long; 0.85–1.12 mm wide at humeri. **Color:** Dorsal integument of head, pronotum, elytra, antenna, and legs various shades of light brown to orange; head and pronotum lighter than remainder; elytral color interrupted by antemedial transverse, white, microstriate, unelevated fascia that does not reach suture; ventral color mostly light brown to orange except for sternites which are brown with very dark brown posterior margins. **Head:** Semi-matte, microsculptured but impunctate throughout; inconspicuous, sparse, translucent long and short erect and suberect setae; frons and gena short, broad, with short, acute projection near base of mandible; with incomplete frontal-genal ridge; without anteclypeal sulcus; without interantennal groove or depression; single eye lobe anteroventrally positioned to antennal tubercle; laterally nearly as protuberant as pronotum; finely faceted; antennal tubercle moderately elevated; antenna 10-segmented, without spines, short, extending to apical third of elytron; scape long, slender, extending beyond anterior fourth of pronotum; antennomere 2 short, but over one-third length of antennomere 3; antennomere 4 distinctly shorter than 3 and 5, 6–10 successively shorter, decreasing in length, produced apicolaterally; antennomeres dark brown with exception of scape which may have light brown base; sparse, elongate, suberect and appressed, white setae throughout. Mandible moderately produced, yellow with piceous apex; terminal palpomeres elongate, not broadly dilated in female; broadly dilated and securiform in male. **Pronotum:** Matte, with uniform ultra-microrugosity throughout, impunctate, without calli or tubercles; distinctly longer than broad, 1.15–1.24 mm long, 0.65–0.88 mm wide (length/width = 1.41–1.76); strongly constricted at basal fourth, elevated and widest anteriorly, base distinctly narrower than elytral base; distinct, rounded periscutellar projection at middle; sparsely but conspicuously pubescent with scattered, long, erect translucent to white setae. **Prosternum:** Glossy, impunctate, with sparse, elongate, white setae; prosternal process very narrow between procoxae; apex broadly expanded behind, closing procoxal cavities posteriorly. **Elytron:** Mostly glossy; impunctate (but with scattered, dark, subcuticular spots resembling punctures but not depressed on surface); microruguse at basal third, with unelevated antemedial, transverse, white, microstriate fascia not attaining suture; oblique, ultra-micropunctate region adjacent and posterior to white fascia; remainder of elytron to apex glossy; scattered, long, translucent setae sparsely distributed throughout; light brown throughout with exception of white fascia which is surrounded by darker brown on both sides, extreme base, and periscutellar regions which are darker brown; weakly gibbous at apex; elytral apex narrowly rounded to suture; 1.97–2.64 mm long, 0.40–0.55 mm wide (length/width = 4.80–4.93). **Scutellum:** Narrow, subtruncate at posterior apex; sparsely coated with appressed, short, yellowish setae. **Legs:** Femora short, stout, with strongly clavate apices on abruptly narrowed peduncles; metafemur not attaining elytral apex; tibiae straight, not expanded apically; meso- and metatibiae each with two asymmetrical, straight tibial spines; protibia with one; tibiae and femora sparsely but conspicuously pubescent with long, erect, white setae. **Venter:** Glossy; sparsely pubescent throughout with erect, long, white setae; dense, white, short, appressed setae present on posterior margin of metasternum to sides, corresponding with white macula of elytron, and along side of mesosternum; integument light brown, but darker on abdominal sternites; mesosternal intercoxal process narrow, but about twice as broad as prosternal process, with strong lateral projection into mesocoxa. Ventrite 1 most elongate; remaining ventrites much shorter and subequal in length; apex of fifth ventrite broadly rounded, without notch, sulcus, or other modification.

**Figure 4.**
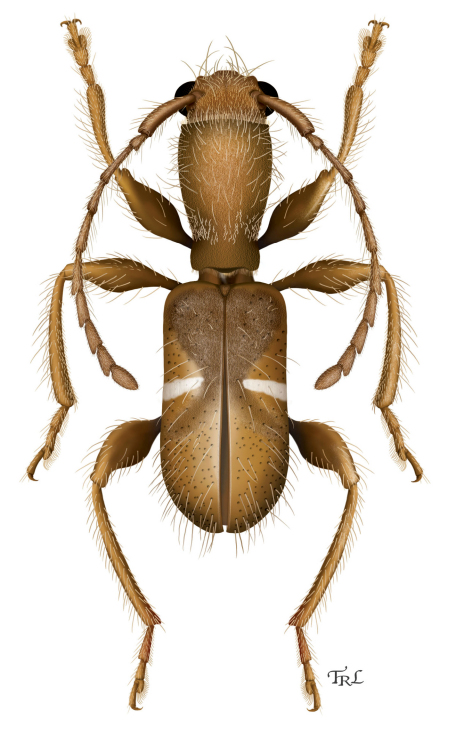
*Tilloclytus baoruco* sp. n., dorsal habitus. Digital painting by Taina Litwak.

#### Etymology.

The specific epithet, a noun in apposition, is based on the mountain range, Sierra de Baoruco, where the holotype was collected.

#### Type material.

Holotype, female: Dominican Republic, Pedernales Prov., Parque Nacional Sierra de Baoruco, Las Abejas, 1150m, beating, E. H. Nearns and S. W. Lingafelter, June 18, 2005 (USNM). Paratypes: Haiti, Dept. Sud-Oueste, Parc National La Visite, ca. 1 km. S Roche Plat, May 22, 1984, M. C. Thomas, collector (FSCA, 1 male); Haiti, Dept. Sud-Oueste, Parc National La Visite, vicinity park headquarters, 1880 m, May 23, 1984, M. C. Thomas, collector (FSCA, 1 male, with associated *Pheidole* sp. ant); Haiti, Dept. Ouest, Furcy, July 9, 1956, B. and B. Valentine, collectors (USNM, 1 male, 1 female; ACMT, 2 females).

#### Remarks.

This species is sexually dimorphic with respect to the terminal labial and maxillary palpi: in males, they are strongly dilated, nearly securiform; in females, weakly dilated and more elongate. One specimen was collected with an undetermined species of ant in the genus *Pheidole* Westwood.

### Tilloclytus neiba

Lingafelter sp. n.

urn:lsid:zoobank.org:act:461DD063-89B4-46D3-812A-30C208D74C8B

http://species-id.net/wiki/Tilloclytus_neiba

Fig. 5 Map 1

#### Diagnosis.

This species is similar to *Tilloclytus rufipes* Fisher from Cuba in proportions, color, and in having the antemedial elytral fascia extend completely to the suture, but in *Tilloclytus neiba* the white elytral fascia is striate and without pubescence; in *Tilloclytus rufipes*, the white fascia is a band of pubescence. *Tilloclytus neiba* also differs from *Tilloclytus rufipes* in having very short, white pubescence covering the entire base of the elytron giving it a matte finish (in *Tilloclytus rufipes*, this portion of the elytron is glossy and mostly free of appressed pubescence). From the Hispaniolan congener, *Tilloclytus baoruco*, *Tilloclytus neiba* differs most distinctly by having 11 antennomeres (10 in *Tilloclytus baoruco*) and in having the white fascia extending to the suture (incomplete in *Tilloclytus baoruco*).

#### Description.

Male. 4.08–4.23 mm long; 1.01–1.25 mm wide at humeri. **Color:** Most of dorsal integument of head, pronotum, and elytra dark brown to golden brown; antenna, legs, mesosternum and sometimes metasternum, prosternum, and base of pronotum, light orange; elytral color interrupted by antemedial transverse, white, microstriate, unelevated fascia that reaches suture. **Head:** Semi-matte, microsculptured but impunctate throughout; covered with moderately dense mixture of short, semi-appressed and long, erect, translucent and golden setae; frons and gena short, broad, with short, acute projection near base of mandible; with incomplete frontal-genal ridge; without anteclypeal sulcus; without interantennal groove or depression; large, single eye lobe anteroventrally positioned to antennal tubercle; laterally as protuberant as pronotum; finely faceted; antennal tubercle moderately elevated; antenna 11-segmented, without spines, short, extending to apical third of elytron; scape long, slender, extending beyond anterior third of pronotum; antennomere 2 short, less than one-third length of antennomere 3; antennomere 4 distinctly shorter than 3 and 5, 6–10 successively shorter, decreasing in length, not produced apicolaterally; antennomeres orange to light (sometimes with 9–11 dark brown); sparse, elongate, suberect and appressed, yellow-translucent setae throughout. Mandible moderately produced, light brown with piceous apex; terminal palpomeres broadly dilated. **Pronotum:** Matte except for glossy posterior fifth and sides, with striate microsculpturing over most of disk; impunctate, without calli or tubercles; distinctly longer than broad, 1.23–1.30 mm long, 0.81–0.84 mm wide (length/width = 1.52–1.55); strongly constricted at basal fourth, elevated and widest anteriorly, base distinctly narrower than elytral base; without periscutellar projection at middle; moderately dense, appressed white to translucent pubescence, especially at anterior third and along posterior constriction, combined with scattered, sparse, long, erect white or translucent setae. **Prosternum:** Glossy, impunctate, with sparse, elongate, translucent setae; prosternal process narrow between procoxae; apex broadly expanded behind, closing procoxal cavities posteriorly; dark brown anteriorly to pale orange posteriorly near procoxae, or uniformly pale orange. **Elytron:** Mostly glossy and impunctate (but with scattered, dark, subcuticular spots resembling punctures but not depressed on surface); inconspicuously micropunctate at basal third with moderately dense patch of yellow-white and translucent, appressed setae combined with more sparse, long, erect setae; with unelevated antemedial, transverse, white, microstriate fascia attaining suture; apical two-thirds mostly covered by patch of moderately dense, short, yellow-white, appressed setae with interspersed long, erect setae; dark brown to light, golden brown throughout with exception of white fascia; weakly gibbous at apex; elytral apex rounded to suture; 2.52–2.56 mm long, 0.51–0.59 mm wide (length/width = 4.33–4.94). **Scutellum:** Broad, short, rounded at posterior apex; moderately coated with appressed, short, yellow-white setae. **Legs:** Femora short, stout, with strongly clavate apices on abruptly narrowed peduncles; metafemur not attaining elytral apex; tibiae straight, not expanded apically; meso- and metatibiae each with two asymmetrical, straight tibial spines; protibia with one broad, curved spine; tibiae and femora sparsely pubescent with long, erect, off-white setae. **Venter:** Glossy; sparsely pubescent with inconspicuous, erect, translucent setae; dense white, short, appressed setae present on posterior margin of metasternum to sides, corresponding with white macula of elytron, and along side of mesosternum; integument light brown to orange on mesosternum and sometimes metasternum; dark brown on sternites; mesosternal intercoxal process narrow, but about twice as broad as prosternal process, with strong lateral projection into mesocoxa. Ventrite 1 most elongate; remaining ventrites much shorter and subequal in length; apex of fifth ventrite broadly rounded, without notch, sulcus, or other modification.

**Figure 5.**
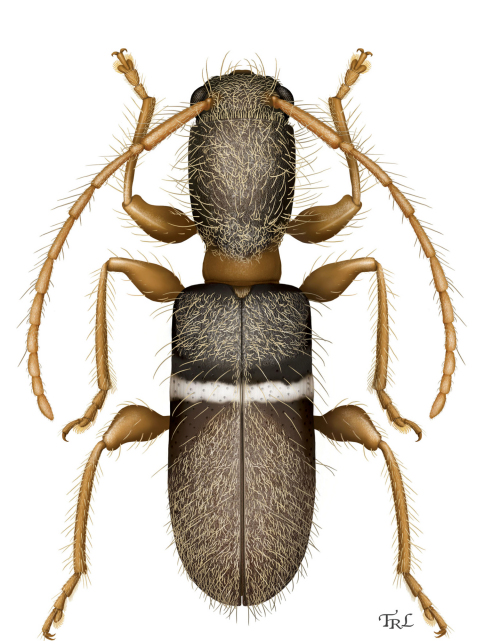
*Tilloclytus neiba* sp. n., dorsal habitus. Digital painting by Taina Litwak.

#### Etymology.

The specific epithet, a noun in apposition, is based on the mountain range, Sierra de Neiba, where the holotype was collected.

#### Type material.

Holotype, male: Dominican Republic, San Juán Prov., Sierra de Neiba, trail to Sabana del Silencio, 10 km SSW of El Cercado, 1650-1700m, 18°39.935'N, 71°31.964'W, July 10-11, 2006, N. E. Woodley, collector, sweeping foliage (USNM). Paratype: Dominican Republic, La Vega Prov., Jarabacoa – El Rio Rd., 910 m, April 11, 1992, M. Ivie, D. Sikes, and W. Lanier, collectors (WIBF, 1 male).

#### Remarks.

Only males are known. Although this species was not collected with ants, *Pheidole* could serve as the model given its similarity to *Tilloclytus baoruco* which was collected with that genus of ant.

## Key to Anaglyptini and Tillomorphini of Hispaniola

There are no worldwide keys to tribes or genera that include all Old and New World taxa of Anaglyptini and Tillomorphini. Linsley (1962) provided a key to the Cerambycinae tribes of North America, but it excluded most of the genera in these groups that occur in the Neotropical Region. The characters that he used to distinguish the tribes (Anaglyptini: “without transverse, ivory-like ridges”; Tillomorphini: “usually with transverse raised ivory-like ridges”) are unsatisfactory (see Introduction). No keys exist to all the species in the West Indies. A key to the three genera of Tillomorphini from the Lesser Antilles is provided in Chalumeau and Touroult (2005). A key to all the Cuban species of Tillomorphini + Anaglyptini is provided by Zayas (1975). In Puerto Rico, only one species in each of the tribes Anaglyptini (*Tilloclytus minutus* Fisher) and Tillomorphini (*Lamproclytus elegans* Fisher) is known, so no keys to the species of those genera were necessary (Micheli 2010). The key below combines all the new species described herein and includes both tribes since there are no satisfactory characters to differentiate them.

**Table:** 

1	Antenna with prominent mesal spines on antennomeres 3–5	*Licracantha formicaria* Lingafelter
–	Antenna without spines	2
2(1)	Antenna very short, extending only to extreme base of elytron; elytron with raised, ivory callus; pronotum uniformly alveolate-punctate	*Calliclytus macoris* Lingafelter
–	Antenna longer, reaching beyond middle of elytron; elytron with unraised, white fascia; pronotum without distinct punctures	3
3(2)	Antenna 10-segmented; elytral fascia not attaining suture	*Tilloclytus baoruco* Lingafelter
–	Antenna 11-segmented; elytral fascia attaining suture	*Tilloclytus neiba* Lingafelter

## Supplementary Material

XML Treatment for Licracantha

XML Treatment for Licracantha formicaria

XML Treatment for Calliclytus macoris

XML Treatment for Tilloclytus baoruco

XML Treatment for Tilloclytus neiba
